# A Sound Education: A Qualitative Study of the Role of Podcasts in Postgraduate Medical Education

**DOI:** 10.1007/s40670-025-02553-y

**Published:** 2026-01-27

**Authors:** Rachel Louise Saville, Steven John Agius

**Affiliations:** 1 University of Nottingham, Nottingham, UK; 2 Middlemore Hospital, Auckland, New Zealand

**Keywords:** Podcast, Medical Education, Postgraduate, E-learning

## Abstract

**Introduction:**

The rise of smart technology has driven increased use of podcasts in medical education due to their accessibility, flexibility, and capacity for rapid dissemination of current information. Existing literature, predominantly North American and undergraduate-focused, is insufficient to understand podcast use and integration into UK postgraduate medical education.

**Materials and Methods:**

Ten Internal Medicine Trainees from the East Midlands region were interviewed online using semi-structured interviews. Recordings were transcribed and analysed using Interpretative Phenomenological Analysis. Ethical approval was obtained.

**Results:**

Four themes emerged: Self-directed Learning, Time Efficiency, Relaxed Learning Environment and Access to Experts. Podcasts have been used informally to address self-identified knowledge gaps and personalise study schedules. Trainees valued concise, high-yield content and the ability to learn while multitasking with other activities such as commuting or cleaning, which was seen as productive. Podcasts user-friendly, enjoyable format created a relaxed learning environment. Additionally, podcasts offered valuable access to expert speakers, modelling clinical reasoning skills, and fostering connections with senior doctors.

**Discussion:**

Trainees actively use podcasts as informal learning tools to supplement their education. The flexibility and enjoyment offered by podcasts support self-directed, continuous learning. Despite regular use, and evidence of their integration internationally, they remain absent from the regional speciality school curriculum. This highlights a missed opportunity for formal recognition, integration and guidance.

**Conclusions:**

Podcasts are a valued but under-recognised educational resource amongst postgraduate trainees. There is demand for their formal inclusion in Continuing Professional Development (CPD) and for educator-endorsed recommendations tailored to training stages.

## Introduction

Podcasts extend the long tradition of learning through speech and storytelling by offering accessible, portable, and on-demand audio education. Their popularity has grown worldwide, especially amongst younger learners [1, 2] and medical professionals [3, 4]. Amongst health professions, physician education is the most extensively studied in relation to podcast use [4, 5], with particularly strong uptake in emergency medicine and critical care [6–8]. Most podcasts are produced by individuals or medical journals, with relatively few linked to universities or training programs [9].

Podcasts supplement medical education, by providing flexibility and accessibility to asynchronous learning material which can be used whilst multitasking [10, 11]. Podcasts have been utilised as a means of learning core clinical content, enhancing understanding of the diagnosis and management of a wide range of medical conditions, supporting examination preparation, reviewing recent literature through journal clubs, and engaging with broader issues such as professional development and burnout [11–17]. Wolpaw [10] highlights a generational gap: while trainees preferred digital, asynchronous tools, Training Programme Directors thought trainees favoured structured didactic methods and assigned resources, underestimating how frequently podcasts were used for self-directed learning. Importantly, when podcasts have been integrated into formal curricula, learners have responded positively [17]. The COVID-19 pandemic has further accelerated the adoption of digital learning formats [18], showcasing podcasts ability to quickly disseminate up-to-date information [19].

Limitations of podcasts include variable content and audio quality [11, 20], passive learning and potential distraction when multitasking with other activities. Substantial evidence shows that multitasking with non-academic internet use, such as texting or social media, impairs learning in classroom settings [21–23], consistent with information processing theory [24], which emphasises the role of attention and working memory. Learners who are metacognitive or self-aware may recognise lapses and revisit missed material to compensate [21]. Despite these challenges, podcasts remain a valuable adjunctive tool. Evidence demonstrates that they deliver knowledge retention comparable to traditional lectures [25] and superior to textbooks [26], and that concurrent activities such as driving or exercising do not diminish knowledge retention [27–29].

Most existing research is quantitative or survey based and concentrated in Canada and North America, with limited qualitative insight into learner experiences. Evidence on postgraduate medical educational podcast use in the United Kingdom (UK) is scarce, leaving questions about their use and integration unanswered.

## Method

The aim of this study was to increase the understanding of the lived experiences of postgraduate medical trainees who use podcasts for the purposes of medical education.

A constructivist, qualitative approach was taken to capture diverse perspectives and uncover the current experience of podcast usage amongst postgraduate learners, addressing a qualitative knowledge gap within the medical education literature.

Interpretive phenomenological analysis (IPA) was chosen for its suitability in exploring lived experiences. IPA, informed by hermeneutics and ideography, acknowledges the influence of the lead researcher’s background as a medical trainee and podcast developer through the process of reflexivity and aligns with the study’s goal to capture postgraduate medical trainees’ experiences with educational podcasts.

### Setting

The study was conducted at the University of Nottingham in 2023 and focused upon postgraduate trainees based in a single region of the UK (East Midlands).

### Sampling Methods

Purposive sampling was used to recruit 10 participants representing a mix of ages, genders, and ethnicities amongst postgraduate doctors in the East Midlands. Participants were Internal Medicine Trainees (IMT) or Specialty Trainees (ST). In the UK, IMT1 marks the first year of specialty training and ST7 or ST8 the final year before becoming a consultant. This phase covers the key exam and professional development period for medical trainees after graduation from medical school. Diversity was sought by recruiting across training stage (IMT1-ST8), gender, and ethnic background, using available demographic information at the time of registration of interest, aiming for an even mix of training stage and self-reported gender, with several ethnicities recruited (Appendix, Table 1). Foundation doctors, consultants, non-medical trainees, and allied health professionals were excluded. The study was limited to the East Midlands for feasibility reasons and because the region hosts *MEMcast*, a podcast founded by the lead author, and created for candidates preparing for the Membership of the Royal College of Physicians (MRCP) postgraduate examinations.

### Recruitment Process

Ethical approval was granted by the University of Nottingham Faculty of Medicine and Health Sciences Research Ethics Committee. Following this, medical trainees in the East Midlands were contacted via email from National Health Service England (NHSE). Ten trainees were recruited and provided with a participant information leaflet and consent form. IPA methodology lends itself to small sample sizes because of the in-depth nature of exploring lived experience of participants.

### Data Collection and Analysis

A semi-structured interview schedule explored participants’ podcast experiences in medical education, collecting demographic data and exploring topics such as podcast discovery, experiences of use, integration into learning and educational impact, and motivations for podcast use. A pilot interview and feedback refined the interview schedule, but the data was excluded from analysis.

Interviews were transcribed using Microsoft Teams software and manually checked for accuracy. Transcriptions were pseudo-anonymised, and videos were deleted post-transcription.

Data analysis followed IPA guidelines: reading transcripts, initial noting, chunking data into themes, identifying relationships, and seeking patterns across cases. Reflective notes documented the researchers’ evolving thoughts and analytical concepts, ensuring reflexivity.

## Results

10 postgraduate trainees participated in the study, ranging from IMT1 to ST7, evenly split between IMT stage 1 (IMT1-3) and stage 2 (ST4-ST8). There were equal numbers of male and female participants and trainees were recruited from across the East Midlands region, with a range of ethnicities. Experiences were gathered from trainees revising for or having completed postgraduate examinations such as the MRCP(UK) and Speciality Certificate Exams (SCE). The age range of participants was 27–37 years.

**Fig. 1:**
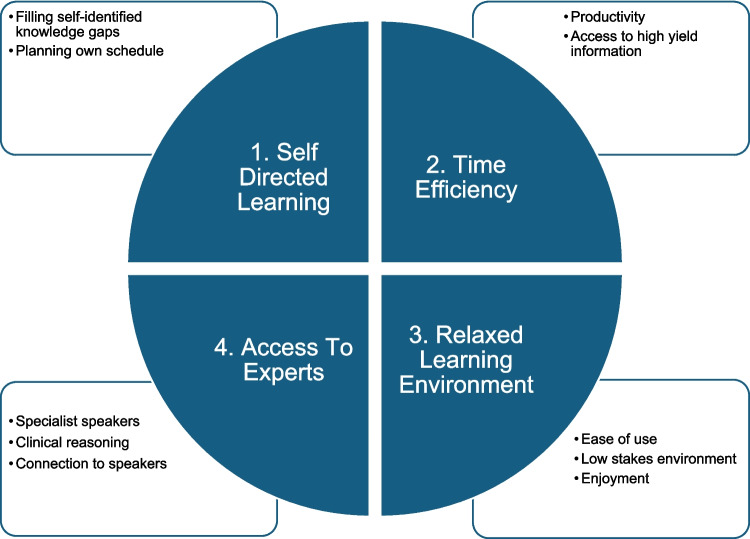
Key Themes Underpinning Podcast Use in Postgraduate Medical Education (PGME). This figure illustrates four superordinate themes that emerged from participant interviews regarding the educational use of podcasts: (1) Self-Directed Learning, (2) Time Efficiency, (3) Relaxed Learning Environment, and (4) Access to Experts

The themes identified (Fig. 1) will be described and supported by verbatim extracts from the case interviews. Each quadrant highlights associated superordinate themes derived from qualitative analysis. These themes reflect the perceived value of podcasts in promoting flexible, efficient and engaging learning experiences for postgraduate medical trainees. All names shown are pseudonyms. The following conventions were used to aid interpretation of the transcripts:

- … pause in speech, break, or omitted text.
- [contextual information or clarification].

### Superordinate Theme 1: Self-directed Learning (Fig. 1)

*‘I think that’s been very self-driven. I haven’t had any formal direction or recommendation from people in senior educational leadership positions, like Training Program Directors or College Tutors, who have said we recommend using this podcast… I think it should be.*’ (Rohan)

Trainees have encountered a lack of formal integration of podcasts into their postgraduate curriculum and received limited recommendations from educators during their structured training. Consequently, they have resorted to utilising podcasts for self-directed learning. Upon identifying knowledge gaps, trainees independently seek out episodes or series that align with their interests and are relevant to their learning needs. This autonomy has empowered them to curate personalised learning schedules, resulting in a tailored learning experience.

#### Filling Self-identified Knowledge Gaps

Some trainees articulated their process of recognising knowledge gaps stemming from recent clinical encounters, limited exposure to certain specialities or conditions during rotational training or missed teaching opportunities. Trainees found podcasts valuable for Continued Professional Development (CPD) and preparation for clinical rotations or work-related events but noted they lacked formal CPD accreditation and expressed interest in such recognition.

*‘Having [General Internal Medicine teaching days] in a podcast form and being able to claim CPD for them would actually make a big difference… sometimes you’re on call and you can’t attend a day.’* (Amit)

*‘I’m usually motivated to go and find [a podcast] episode by a clinical encounter that I feel I could have perhaps handled better, or [when] I felt my approach could have been more robust, or my knowledge in that area could have been… better.’* (Rohan)

*‘You essentially get 6 rotations to get a General Medical experience, and there are, you know, what is it, 50 medical specialties?… I know an hour of an expert talking isn’t the same. You know I don’t claim that that’s equivalent to a four-month rotation, but listening to that I think would go some way and kind of redistributing that experience I think.’* (Ravi)

Increased familiarity with a subject led to increased confidence at work, as trainees felt more prepared for rotations and clinics. Emily described returning from maternity leave and using podcasts to target her own personal learning needs when the formal teaching provided may not fit her personal learning agenda:

*‘It’s kind of about feeling prepared, like knowing… what sort of things I’ve got coming up at work. And then I can try and sort of prepare a little bit in terms of knowledge for it.’* (Emily)

#### Planning own Schedule

Trainees were able to discover podcasts tailored to their current stage of training, enabling them to curate a personal library of resources that align with their specific goals. Whether prioritising exam preparation or skill development, delving into career options, seeking to solidify their understanding, or simply exploring a personal interest, podcasts are viewed as catering to diverse learning needs.

*‘That’s kind of the main advantage… [it] can be guided by you as the listener a little bit because you can say this is what I want to learn about.’* (Emily)

Creating a library of content highlights resources for later revision. This ‘on demand’ learning is viewed as easily accessible and flexible, allowing trainees to control their schedule, improving concentration and focus.

*‘Before a rotation, it’s almost like habit to just look up like some podcasts or… some that I have in like my favourite list.’* (Asha)

*‘During MRCP… if I was doing a cardiology week, I would listen to the Cardiology podcast or the PACES [Practical Assessment of Clinical Examination Skills] one, if I was doing Respiratory week, I would do that. So that’s how I chose what podcast to listen to, based on my own revision.*’ (Priya)

### Superordinate Theme 2: Time Efficiency (Fig. 1)

A limited time capacity for learning as a postgraduate trainee was highlighted due to high workloads, busy work schedules, and a wish to maintain a work life balance whilst revising for postgraduate exams and engaging in CPD, meaning that trainees must use the time available to them productively and efficiently with high yield resources.

#### Productivity

Charlotte describes adjusting to life as a postgraduate trainee and the challenges of limited time for personal study when compared to undergraduate training:

*‘You know, when you’re revising for an exam, you’re not just revising for an exam like you were at medical school… you had set time to sit and revise, and that was almost your job... Whereas now being a postgrad[uate] trainee, you’re working at least nine to five, plus long days, plus weekends, plus nights. Actually having the time to revise, for an important exam, that’s actually crucial to your career development, is really hard... So I think it’s really important to try and maximise the revision you can do in the small time that you have [be]cause you don’t want your revision to be spilling then out into your social life. I mean it does, doesn’t it?... If you can revise whilst doing something else like driving to work, you’ve then saved yourself a bit of time, perhaps in the evening that you can spend doing something not medical and actually just living your life.’*

Unable to access protected time at work, trainees use multitasking to feel productive. They recalled cleaning, cooking, exercising, travelling, and engaging in hobbies whilst listening to podcasts, however the most consistent use was during commuting. As trainees rotate around a variety of hospitals in a particular region during training, they often commute. Utilising time efficiently by multitasking helped trainees feel productive, using otherwise ‘wasted time’ and maintain a work-life balance.

*‘I have a lot of anxieties about wasting time… We’re put in places where we have to commute… and we’re always revising for these things in our spare time. So I tried to come up with strategies that kind of minimises wasted time… Podcasts, especially for driving, especially when you’re doing something else, it’s just you can do two things at once so easily… You’d feel less like you’ve wasted that hour when actually you’ve gained something out of it.’* (Ravi)

*‘[Podcasts are] easy to listen to, like I can listen to them when I’m crocheting… or when I’m cleaning or doing laundry or doing something that doesn’t require me to, like, focus on what I’m doing.’* (Asha)

Doing simpler tasks or driving a regular commuting route required less ‘brain power’ than other activities and so participants felt they were able to listen and concentrate more on the podcast.

#### Access to High Yield Information

The podcasts used by trainees were generally short, less than one hour, and contained high yield information in the form of summarised papers, guidelines, clinical top tips, and topic overviews. They found this efficient, as trainees did not need to spend time finding, reading, and assimilating raw original literature themselves:

*‘I don’t need to go and find 20 journals, I can just have an expert in the field talk to me about all the evidence in a summarised way.’* (Priya)

*‘I probably wouldn’t have the time to go through all those papers and read and go to all these conferences*’ (Amit)

### Superordinate Theme 3: Relaxed Learning Environment (Fig. 1)

The informal, easy to use and entertaining format allows learners to absorb information at their own pace without the stress of formal settings, facilitating a more enjoyable and relaxed learning experience.

#### Ease of Use

Participants found podcasts were user-friendly, convenient, flexible and more digestible compared to other resources. Trainees acknowledged the passive nature of learning through podcasts, recognising the low effort required. Subscriptions made it easy to find content. Generally, trainees tended not to engage in active learning techniques while using podcasts. Activities such as note-taking, pausing to answer questions, or completing recommended follow-up tasks although reported, were infrequent. The preference leaned towards a more relaxed approach to consuming podcast content.

*‘You come home from work one day and you want to read up about a case that was interesting, but you don’t really have the energy… I often… listen to a few podcasts… it is again a very low effort but high reward.’* (Priya)

*‘I guess podcasts help because I feel like I have done revision without trying so hard. So I’m not sitting down and saying revise neurology for two hours I think ahh I’ll just put a podcast on doing something else. So it feels a bit like less intense.’* (Olivia)

#### Low Stakes Environment

Podcasts are less intimidating than face to face events where learners may be asked to answer questions in front of peers, or they may struggle to keep pace with the lecturer. They also create a more relaxing method of revision when preparing for high stakes examinations, allowing trainees to revisit specific parts of the podcast if their focus drifted or when grappling with unfamiliar concepts. There was no expectation for immediate comprehension:

*‘I don’t have to get it the first time. I just have to listen to it and there’s no pressure. Like, it’s not like I go to a lecture and they’re gonna ask me questions about it… I like to listen to a podcast first, because sometimes I find it quite daunting to read about a topic.’* (Asha)

*‘That’s the joy of it… if you’ve played the whole episode and then you’re like, oh, actually, I’ve only listened to like 10% of that because my mind’s been thinking about something else, then you can just listen to it again...’* (Emily)

#### Enjoyment

Trainees enjoyed listening to podcasts which were less mundane than conventional materials, often featuring engaging elements like entertaining twists, games, or musical interludes. The conversational tone of the podcasts contributed to a laid-back and pleasurable listening experience, even for those utilising them to prepare for critical examinations:

*‘I found that podcast particularly relaxing, and it was different. It was nice because at the time I was quite stressed [about exams] and it was still medical, and that was nice or like relevant, so I didn’t feel guilty.’* (Asha)

### Superordinate Theme 4: Access to Experts (Fig. 1)

Podcasts provide trainees with access to expert speakers, compensating for potential gaps in local teaching and learning opportunities. They offer a valuable avenue for acquiring both knowledge and clinical reasoning skills. Trainees highlighted a sense of connection with the speakers, relating to them through shared experiences and acknowledging their fallibility.

#### Specialist Speakers

Trainees were interested in accessing podcasts that involved conversations with experienced clinicians, particularly registrars, consultants, and doctors engaged in research. This interest stemmed from the speaker’s ability to simplify complex subjects and the ability to access experts that trainees may not readily encounter through conventional training:

*‘There’s a lot of heterogeneity in what services [are] available in each place and with our rotational training, we’re not exposed to every single thing. So in Parkinson’s… I have never seen [deep brain stimulation] and I don’t think I’ll ever see that in my career. And I’ll probably never meet an expert who’ll speak to me about it… and having a podcast where they explain these things… and why they do it is just such a… easy to access resource.’* (Ravi)

#### Clinical Reasoning

Trainees found value in listening to the thought processes of specialists, as they made their clinical reasoning explicit. The authenticity of the cases discussed, and the transparency of the experts’ thinking were highly regarded. Consequently, trainees identified the ability to replicate these critical thinking skills in their own clinical practice.

*‘As I’ve progressed through training, I’ve realised that actually I do need to know the knowledge… But the way people think is also quite useful, like how a neurologist thinks about neurology… How the Endoscopist thinks about an upper [gastrointestinal] bleed.’* (Asha)

#### Connection to Speakers

The trainees feel a sense of connection with the podcast hosts, guests, and other listeners, feeling like they are involved in discussions, and developed a familiarity and comfort with the regular hosts:

*‘You feel like you’re having a personal chat with this other person that feels like they’re talking to you, and I like that social aspect of it. You can’t really get that if you just read a book.’* (Priya)

The candid discussions by experts about their experiences contribute to humanising and making them relatable. These insights convey the message that one does not need to possess an exhaustive understanding to practice medicine. This realisation promotes a more approachable and attainable perspective for trainees, inspiring confidence as they navigate their own paths in the medical field:

*‘It’s also quite encouraging when they sometimes don’t know… And I’m like, oh, that’s okay. You don’t know everything.’* (Olivia)

*‘They talk about not only things that may have been interesting and gone well, but things where they’d made mistakes as well… we’re probably not alone… when we look after people with a great deal of uncertainty and we’re not sure if we’ve done the right thing or not’* (Zayn)

## Discussion

This study provides new insights into how postgraduate medical trainees in the East Midlands use podcasts for medical education. While previous research has explored digital learning tools, this study highlights the unique role of podcasts in bridging gaps in traditional PGME. The findings align with self-directed learning theory [30], emphasising that trainees actively seek flexible and time-efficient learning strategies to navigate their demanding schedules.

Prior studies have demonstrated the effectiveness of podcasts in supplementing medical education, particularly in the United States and Canada, or undergraduate settings [31–34]. However, their role in British PGME remained underexplored. Our findings suggest that podcasts serve as a critical adjunct to formal education, enabling trainees to access expert insights and clinical reasoning beyond structured teaching. This resonates with social learning theory [34], which emphasises observational learning — trainees benefit from hearing experts articulate their thought processes, something often limited in traditional didactic settings.

Despite these advantages, our study also reveals a lack of formal integration of podcasts within PGME in the East Midlands. While other regions [4, 17] have successfully embedded podcasts into structured curricula, the reliance on informal, word-of-mouth recommendations in our cohort limits their potential impact. This highlights an opportunity for institutions to curate high-quality podcasts and provide formal recommendations, enhancing the consistency and credibility of podcast-based learning.

Our findings have important implications for medical educators and policymakers. Prior research has demonstrated that podcasts can support both the acquisition and retention of medical knowledge [26, 35–38] as well as the development of skills [39, 40]. While participants in this study reported frequent podcast use and a strong appetite for recommendations and CPD accreditation, their educational value is likely to be maximised only if educators provide structured guidance toward high-quality, evidence-based content. This underscores a gap in current educational practice: acknowledging learner preferences is insufficient unless paired with deliberate strategies from educators to curate, quality-assure, and embed such resources into postgraduate curricula.

In addition, the capacity of podcasts to facilitate learning during multitasking activities, such as commuting or exercise [27–29], raises important considerations for PGME. Used alongside traditional learning methods, the flexibility of podcasts offers valuable opportunities to align education with the demanding schedules of trainees. If leveraged thoughtfully, asynchronous and mobile-friendly educational strategies could not only enhance accessibility but also contribute to mitigating stress and burnout amongst trainees.

Moreover, the preference expressed for shorter podcast episodes (under one hour) is consistent with existing research on cognitive load and attention span in adult learners [11, 41–43]. While brevity could prevent cognitive overload, there is a risk that excessive simplification may compromise depth of understanding. Educators and content creators should therefore aim to design episodes that strike a balance between conciseness and substantive content, ensuring that efficiency does not come at the expense of educational rigor. From a curriculum design perspective, shorter podcasts may be most effective when integrated as supplementary resources that reinforce or consolidate key concepts, rather than as stand-alone replacements for more comprehensive instructional methods.

The limitations of this study must be acknowledged. The study’s geographical scope restricts generalisability, as podcast engagement may vary across regions, specialties and countries. Additionally, requiring active podcast use for participation may have introduced selection bias, favouring trainees with positive attitudes toward podcasts (Appendix Table 1).

## Conclusion and Recommendations

This study addresses a critical gap in the literature by examining the role of podcasts in PGME within the UK. Findings suggest that while podcasts serve as a powerful supplement to traditional learning, their full potential remains untapped due to the lack of institutional endorsement. To optimise learning outcomes, PGME programs should:

1. Formally integrate podcasts into educational frameworks.
2. Provide structured guidance on effective podcast use, ensuring trainees benefit from credible, well-produced materials.
3. Encourage podcast creators to align content with medical curricula and best practices in adult learning theory.

Trainees highlighted the value of gaining insight into the "way of thinking" of experts, which emerged as a key superordinate theme in the study. Future research should quantify the impact of podcasts on clinical reasoning and decision-making skills and explore their long-term effects on professional development. Podcasts offer a unique opportunity for trainees to access expert perspectives and clinical reasoning in a flexible, on-demand format. Understanding how these resources influence trainees’ ability to think critically and make informed decisions in clinical practice could have significant implications for their professional development. Future studies should explore podcast use across different specialties and regions in the UK to provide a broader understanding of their impact on medical education.

Given their accessibility and effectiveness, podcasts could play an increasingly central role in modern medical education, helping bridge the gap between structured training and real-world clinical challenges.

## Data Availability

Data is stored in the Nottingham Research Data Management Repository (https://rdmc.nottingham.ac.uk) and is available on request.

## Appendix

**Table 1:** Demographic profile of participants

Participant pseudonym	Gender	Age	Ethnicity	IMT Stage 1 or 2	North or South trainee	Exam status
Amit	Male	30	Indian	Stage 2	North	Post MRCP and SCE
Zayn	Male	30	British Asian	Stage 2	South	Post MRCP
Emily	Female	37	White British	Stage 2	North	Post MRCP
Ravi	Male	30	British Asian	Stage 1	South	Mid-MRCP
Charlotte	Female	28	White British	Stage 1	South	Post MRCP
Ali	Male	36	British Pakistani	Stage 2	South	Post MRCP and SCE
Priya	Female	33	British Asian	Stage 2	North	Post MRCP
Olivia	Female	27	White British	Stage 1	North	Mid- MRCP
Rohan	Male	29	British Asian	Stage 1	South	Mid-MRCP
Asha	Female	28	Sri Lankan	Stage 1	North	Post MRCP

## Interview Schedule

I. Opening
  1. (Establish Rapport) Hi, my name is Rachel and thank you very much for agreeing to take part in my research study for my Masters Dissertation Project at the University of Nottingham.
  2. (Purpose) I would like to ask you some questions about your background and education, some experiences you have had using podcasts in your postgraduate life. I would like to learn more about your lived experiences and share this information in my dissertation and hopefully subsequently, publish the data.
  3. (Motivation) I hope to use this information to help resource developers and curriculum designers understand how podcasts are currently being used amongst postgraduates in our region, to use when creating podcasts and when writing curricula or teaching programmes.
  4. (Time Line) The interview should take between 45–60 min. Are you available to respond to some questions at this time?
  5. I will not be using your name throughout this interview to protect your identity in the transcripts, if names are used they will be removed from the transcripts before data analysis. Data will be anonymised in subsequent reports or publications, and all data will be stored securely as per the data management plan which can be provided on request.
    After I start the recording, I’m just going to ask you again if you are happy to take part in the study, so I can record your consent.
    START TRANSCRIPTION AND RECORDING
    Thank you again for agreeing to be part of this study titled ‘How are podcasts used in postgraduate medical education’. We spoke briefly before starting the recording. Can you confirm that you have read the patient information sheet, and completed the consent form?
    (Yes)
    If at any point, you wish to terminate the interview, please let me know and we can stop the recording.
    If, after the interview has completed, you wish to withdraw from the study, please let me know within the next 7 days. After this time, already anonymised data will not be able to be removed as it will have been incorporated into the study.
    Do you have any questions before we start?
    Could you please confirm your consent again for the recording?
    (Yes)
    Thank you.
    Transition to the next topic: Let me begin by asking you some questions about you so I know a little more about you and understand where you are in your training.
II. Body
  A (Topic) General demographic information/Education
    1. Could you start by telling me your gender, age, and ethnicity?
    2. And can you tell me about your medical training and qualifications to date?
      Prompts:
      - What stage of Internal Medicine (IM) training are you at (IMT1-8)?
        - ◦ For 1–3
          - ▪ Do plan to apply for a particular registrar specialty?
          - ▪ Do plan to continue to train in IM as a registrar?
        - ◦ For 4–8
          - ▪ Are you still dual training in IM?
          - ▪ What other speciality are you training in?
      - What *postgraduate* exams have you sat so far?
      - What *postgraduate* exams do you plan to sit in the future during training?
      - Are you preparing to sit an exam soon?
      - Have you always trained in the East Midlands?
        Transition to the next topic: Ok thank you for that. Since you have enrolled in this research, you said that you have listened to podcasts for medical education purposes in the last 6months. I want to ask you about how you find podcasts to listen to…
  B (Topic) Discovery
    1. Can you tell me about how you might find and then continue engaging with a new educational podcast?
      - PROMPTS: Anything else? > Finding new episodes/shows: (eg word of mouth, social media, teachers, deanery email, google search, podcast platform search etc.?
      - How does that compare to finding non-educational podcasts?
      - What influences your decision to listen to a new podcast?
      - How long would you listen to a podcast before you decide that it is/isn’t for you?
      - Are there occasions where you do not listen to a full episode? Why not?
      - What would make you recommend a podcast to a friend?
        Transition to the next topic: Thank you. Next, I wanted to ask you about your motivations for using podcasts for medical education…
  C (Topic) Motivation
    1. What are your perceived benefits and motivations for using medical educational podcasts?
      - PROMPTS: Any other reasons?
        - ◦ Eg CPD/Exams/revision/Entertainment?
          Transition to the next topic: Thanks for sharing that. Next, I’d like to ask you about your experiences listening to podcasts.
  D (Topic) Experiences of podcast use 
    1. How did you use your most recent Medical Education podcast?
      PROMPTS:
      - Which podcast did you listen to?
      - When was the last time?
      - How often do you listen to educational podcasts (High/mod/low user?)
      - What time of day do you listen?
      - What are you doing as you listen to a podcast?
        - ◦ Eg Multitasking? (driving, exercising, chores) Sat at desk/making notes, focused activity
        - ◦ Do you listen through first time and then second make notes?
      - What is the format of your favourite medical education shows and why?
        - ◦ How are the podcasts that you listen to structured? (mention earlier series volunteered by participant)
          - ▪ Eg Unedited complete lectures; short explanations of difficult concepts; assessment explanations, feedback provision, and suggestions for further reading
        - ◦ Do the podcasts you listen to give you follow-up tasks/activities?
          - ▪ If so, do you complete them?
          - ▪ If they don’t, but they did, would you do them?
      - What facilitated the experience, and what could have been better?
      - How do you physically listen to podcasts?
        - ▪ Where are you? Library, home, commute, outside etc.
        - ▪ Accessed via what device: laptop/smartphone/smart speaker etc.
        - ▪ In series? Pick and choose topics? Playlist?
        - ▪ Regular listener, or in chunks before test/exam
        - ▪ Do you repeat episodes – if so, on average how many times? Do you save sections?
        - ▪ Do you ‘favourite’ episodes to relisten to?
        - ▪ Download or stream?
        - ▪ Online/offline?
        - ▪ Alone/groups?
      - How much attention would you say you gave to the podcast?
        If not the same way of discovery as above, ask earlier you said that typically you use xxxx to find podcasts. Can you tell me why you think you selected this podcast differently?
    2. How do your personal experiences as postgraduate medical trainee in the East Midlands influence your use of educational podcasts?
      - PROMPTS: How do podcasts fit into your work and home life?
        - ◦ (exams/family life/commuting/busy on calls/stresses of PG training and work)
        - ◦ Do you choose a podcast for a particular focus? I.e. if medical speciality – just medical speciality podcasts or used to broaden medical knowledge eg surgery/paediatrics?
        - ◦ Is there a relationship between the timing of the podcast episode and a learning event? ‘just in time’ learning/After events when you’ve found an interesting case?
      - What challenges do you encounter when using podcasts?
      - Do you feel part of a podcast community?
        - ◦ Is listening to a podcast individual activity? Or group discussions after? (eg book club)
        - ◦ Why/why not?
    3. How has your use of educational podcasts developed during your postgraduate training?
      Transition to the next topic: Next I want to ask you about how podcasts are integrated in to your learning…
  E (Topic) Integration and impact on education 
    1. How do you feel podcasts have been integrated into your learning processes?
      - PROMPTS: How do you use podcasts with other resources?
      - How do you typically access online/offline resources when revising?
        - ◦ Eg I like to block out an hour in my bedroom/go to the library, phone off, cup of coffee, do not disturb etc.
      - What challenges could learners have to integrate podcasts into their learning?
        - ◦ Do you have any examples of personal experience?
      - How do you feel podcasts have impacted your education?
      - How are podcasts integrated into your formal teaching?
        - ◦ If not, why do you think that is?
        - ◦ Prompts: Pre-reading? Post lesson ‘additional reading’
        - ◦ Have podcasts ever formed a mandatory part of a course or formal teaching activity?
          - ▪ Can you tell me about that?
        - ◦ What challenges do you perceive educators may have at integrating podcasts into the curriculum?
      - If you have PG training in another region, has this integration been different in other training areas? If so, how?
    2. In what circumstances would you prefer a podcast over other learning resources?
III. Closing
  A Well, it has been a pleasure finding out more about you and your experiences, thank you. Do you have anything else you think is relevant to the question ‘How do you use podcasts in PG Medical education?
  B (Action to be taken) I should have all the information I need. Would it be alright to email you again if I have any more questions? Thanks again.
